# New Antioxidant Active Packaging Films Based on Yeast Cell Wall and Naphtho-γ-Pyrone Extract

**DOI:** 10.3390/polym14102066

**Published:** 2022-05-18

**Authors:** Guillermo D. Rezzani, Elodie Choque, Andrés G. Salvay, Florence Mathieu, Mercedes A. Peltzer

**Affiliations:** 1 Laboratory of Obtention, Modification, Characterization and Evaluation of Materials (LOMCEM), Department of Science and Technology, University of Quilmes, Bernal B1876BXD, Argentina; asalvay@unq.edu.ar (A.G.S.); mercedes.peltzer@unq.edu.ar (M.A.P.); 2 National Scientific and Technical Research Council (CONICET), Buenos Aires C1425FQB, Argentina; 3 BIOPI-UPJV Laboratory UMRT BioEcoAgro INRAE1158, Université de Picardie Jules Verne, SFR Condorcet FR CNRS 3417, UFR de Sciences, 33 Rue Saint Leu, 80000 Amiens, France; 4 Laboratoire de Génie Chimique, Université de Toulouse, CNRS, INPT, UPS, 31326 Toulouse, France; florence.mathieu@toulouse-inp.fr

**Keywords:** yeast cell wall, naphtho-γ-pyrones, antioxidant, bioactive films

## Abstract

The main objective of this work is the development of new active films based on yeast cell wall obtained by high-pressure homogenization (YCW-H) supplemented with naphtho-γ-pyrone (CL-NGP) extract, which is a bioactive compound produced by Aspergillus tubingensis G131 with great antioxidant potential. A complete characterization of the functional properties of the bioactive films, such as their structural, colour, thermal, mechanical, hydration and water vapour transport, was carried out to evaluate the influence of the addition of the antioxidant compounds. Likewise, the antioxidant capacity of the developed materials and the specific migration of NGPs in food simulants were evaluated. The results showed that CL-NGP extract possessed an important antioxidant activity, which was maintained after incorporation in YCW-H films. The addition of 2 and 5% CL-NGPs decreased the hydration of films and consequently improved the water vapour barrier properties. It was observed that CL-NGPs migrate in fatty food simulants and retain their antioxidant capacity in the simulant. The results obtained in this work showed that bioactive films based on yeast cell walls with the addition of CL-NGPs have the potential to be used as packaging material in systems of interest in the food industry.

## 1. Introduction

To reduce oxidation in sensitive food products, the addition of antioxidants and the design of a suitable vacuum or modified atmosphere packaging technology are the two most common alternatives. However, innovative materials such as the design of active antioxidant packaging systems are being developed [1]. These active packagings prevent oxidation either by absorbing components that contribute to oxidation, such as oxygen or radicals, or by releasing antioxidants within the container, particularly to the surface where the oxidation process mainly occurs. Natural antioxidants of plant and herbal origin are mainly used for these purposes. Oregano (*Origanum vulgare* L.) and thyme (*Thymus vulgaris* L.) have been extensively studied for their antioxidant activity, due to the high content of phenolic compounds: carvacrol and thymol and their incorporation into polymer matrices to develop the active systems were highly described [2,3,4]. To meet the high demand for natural products, manufacturers are constantly looking for new natural molecules with useful properties that could replace or decrease synthetically produced chemicals in their products. In this search for new natural compounds, fungi and bacteria are potential resources of active compounds due to their diversity and production of secondary metabolites with different biological activities [5]. Naphtho-γ-pyrones (NGPs) are molecules within the group of secondary metabolites that are beneficial for health and present already-proved antioxidant characteristics [6]. These metabolites are produced by a wide variety of filamentous fungi, lichens [7], some plant varieties [8], and echinoderms [9]. Furthermore, these compounds have a wide range of biological activities such as antioxidant, antimicrobial or antitumor activities [10,11,12]. Studies carried out by Choque et al. showed that *Aspergillus tubingensis* G131, isolated from a French Mediterranean vineyard, is a good candidate for producing NGPs, and also that there is no production of mycotoxins [13]. NGPs are present worldwide as monomers or dimers, and depending on the structure of the molecule, the radicals that compose it, and the orientation of these radicals, NGPs possess different biological properties of interest for the food and medical industries [14]. The most antioxidant NGP is a dimer presenting numerous free hydroxyl groups [14]. The use of NGPs as antioxidants in food was reported by Zaika and Smith, but the use of these antioxidants in active systems has not been explored so far; therefore, the study of these systems is considered to be of special interest [15].

On the other hand, the use of biopolymers as substitutes for non-degradable traditional plastic is considered a sustainable alternative, particularly interesting for short-term applications, such as food packaging [5,16]. The natural polymers used to develop biodegradable materials include polysaccharides, proteins, and lipids. Studies carried out on polysaccharide films, protein films, and mixes of both showed good properties for the formation of matrices intended for food products’ contact [17]. Biomass of baker yeast, Saccharomyces cerevisiae, has demonstrated the potential of this matrix to form films thanks to its composition of proteins and polysaccharides (β-glucan) [18]. On the other hand, the yeast cell wall, composed mainly of β-glucans and mannoproteins, has also shown good film-forming capacity with characteristics different from those obtained by total yeast biomass [17,19]. In particular, the films formulated from the yeast cell wall have less hydrophilic characteristics and a greater barrier to water vapour, compared to the films obtained from the total biomass [19]. Crude and purified NGP extracts were also tested as active compounds in enzymatically hydrolysed yeast cell wall-based films [6]. Likewise, the use of the yeast cell wall is in line with the interest in the reuse and recovery of industrial waste, since it is part of the waste from the production of yeast extract, as well as from the beer industry [20]. In this way, it would be important to obtain the yeast cell wall through a simpler and more scalable process, such as high-pressure homogenization.

Therefore, the main objective of this work is the development of new biodegradable active films based on yeast cell wall obtained by high-pressure homogenization (YCW-H) and an extract of NGPs obtained in chloroform (CL-NGPs) and re-suspended in anhydrous ethanol after smooth drying. Bioactive films were fully characterised in order to understand the influence of CL-NGPs on YCW-H film properties and the potential to produce innovative antioxidant materials.

## 2. Materials and Methods

### 2.1. Preparation of Yeast Cell Wall by High-Pressure Homogenization (YCW-H)

A 10% *w*/*v* dry base (d.b.) dispersion of commercial bakery yeast (AB Mauri, Buenos Aires, Argentina) was prepared in distilled water. The dispersion was submitted to high-pressure homogenization process (high-pressure homogenizer Panda 2K NS1001L, GEA Niro-Soavi, Parma, Italy) at 125 MPa for 9 min to break the cell, releasing the cytoplasmic material and separating it from cell wall. Subsequently, the homogenized dispersion was centrifuged at 12,000 rpm at 4 °C for 15 min (Avanti Centrifuge, JA-14 Rotor, Beckman Coulter, Brea, CA, USA). The precipitate (YCW-H) was washed three times with distilled water and successive centrifugations under the conditions described above. The solids content was determined at 105 °C until constant weight.

### 2.2. Preparation of NGP Extract

Sporulation of *A. tubingensis* G131 was produced following the procedure of Choque et al. [6]. The NGPs were obtained by solid–liquid extraction using chloroform as extraction solvent. For this, the sporulated mycelium was placed in a beaker with 50 mL of chloroform covering it completely. It was incubated for 20 min at room temperature and then submitted to sonication for 20 min at 50 Hz. The mycelium was filtered on Whatman 113 V paper and an amber extract was obtained. Subsequently, the chloroform was evaporated and the solid residue was suspended in anhydrous ethanol (Anedra, Research AG SA, Buenos Aires, Argentina). This ethanolic extract was identified as CL-NGPs and contained 90% NGPs [6].

### 2.3. Preparation of Bioactive Films

For the preparation of the bioactive films, a dispersion of 5% *w*/*w* (d.b.) of yeast cell wall was prepared. The pH of the dispersion was adjusted to 11 with 0.1 M NaOH solution and a heat treatment at 90 °C was performed for 20 min in a water bath. To break agglomerates produced during heating, the dispersion was treated with Ultraturrax at 12,000 rpm for one minute. The CL-NGP extract was added to prepare formulations: 0, 1, 2 and 5% *w*/*w* (d.b.), matching the amount of ethanol added in all formulations. Then, 25% *w*/*w* of glycerol (d.b.) (Biopack, Sydney, Australia) was added as a plasticiser. To obtain films of thicknesses close to 0.12 mm, volumes of 15 mL of film-forming dispersion were placed in each plastic Petri dish with 86 mm of diameter. Films were obtained by casting at 40 °C for 15 h in a ventilated oven until the remaining water content of the films was 10%. Then, the films were conditioned to 43% relative humidity (r.h.) for subsequent characterization. These films will be identified as YCW-H and their corresponding CL-NGP concentration.

### 2.4. Bioactive Films Characterization

#### 2.4.1. Thickness and Density Measurements

For density determination of dried films, samples with a circular area of 58 cm^2^ were dried at 0% r.h. into desiccators containing silica gel until a constant weight was achieved; this process lasted around 7 days. Films were weighed using an analytical balance (±10^−4^ g). Film thickness was measured by a digital micrometer (±10^−6^ m) at fifteen different places of the film, obtaining for each specimen an average value of 0.12 mm with an error lower than 5% error. Film density of dried film $\rho_{d.f.}\;$(g m^−3^) was calculated through Equation (1):

(1) $$\rho_{d.f.}=m/\left(AL\right)$$

where *m* is the dry mass (g), *A* the area (m^2^), and *L* the thickness (m).

#### 2.4.2. Thermal Properties

The thermal stability of the samples was performed in an inert atmosphere of nitrogen (60 mL/min) using a TGA Q-500 device (TA-Instruments, New Castle, DE, USA). Films were previously conditioned at 43% r.h. Approximately 10 mg of each sample were weighed in a platinum pan and heated from 30 to 100 °C, at 1 °C min^−1^, to study in detail the loss of hydration water. Then, samples were heated at 20 °C min^−1^, from 100 to 600 °C, to study the thermal degradation of the films. The initial degradation temperature (T_ini_) was calculated as the temperature at which 15% of the mass was lost and the maximum degradation rate (T_max_) at the peak temperature determined in the derivative curve (DTG). Experiments were carried out in duplicate.

#### 2.4.3. Fourier Transform Infrared Spectroscopy (FTIR)

The tests were carried out using Shimadzu IR-Affinity infrared spectrophotometer (Shimadzu Co., Beijing, China) equipped with a diamond-tipped ATR module (GladiATR, Pike Technologies, Fitchburg, WI, USA). The spectra were obtained in duplicate in the range of 4000 to 500 cm^−1^, 48 scans and a resolution of 4 cm^−1^. A blank spectrum in air was obtained before each test to compensate the humidity effect and the presence of carbon dioxide.

#### 2.4.4. Mechanical Uniaxial Tensile Tests

Tensile tests were performed on a Universal Test Instrument Megatest^®^ TC-500 series II (Micrometric SRL, Buenos Aires, Argentina). Films were cut into strips with a test dimension of 10 mm × 50 mm and stored until analysis in a controlled atmosphere of relative humidity of 53% given by a saturated solution of Mg(NO_3_)_2_. Tensile tests were performed using a 30 N load cell, with an initial gap between jaws of 25 mm and a tensile speed of 5 mm/min. The percentage of deformation until rupture (ε, %), elastic modulus (E, MPa), and ultimate tensile strength (TS, MPa) for each film composition were calculated from the resulting stress-strain curves as an average of five measurements (*n* = 5) according to ASTM D882 [21].

#### 2.4.5. Colour Determination (CIELab Coordinates)

The change in the colour of the samples due to the addition of the CL-NGP extract was determined using a Konica Minolta CR-400 model. Films were placed on a white surface and the CIELab space was used to determine the parameters L*, a*, b* and Δ*E*, where L* corresponds to the luminosity or darkness of a colour, a* value corresponds to the green to red colour (−a* = greener, +a* = redder), and b* to the blue to yellow (−b* = bluer, +b* = yellower). The colour change ∆*E* was calculated through Equation (2):

(2) $$\Delta E=\sqrt{{\left(L^{*}-L_{o}\right)}^{2}+{\left(a^{*}-a_{o}\right)}^{2}+{\left(b^{*}-b_{o}\right)}^{2}}$$

where $L_{o}$, $a_{o}$ and $b_{o}$ are the coordinates corresponding to the blank [22]. Five points were measured for each formulation.

#### 2.4.6. Measurement of Antioxidant Activity by the ABTS Method

Radical solution of ABTS preparation: the radical solution of ABTS (ABST^•+^) was generated by reacting 7 mM solution of 2,2’-azinobis-(3-ethylbenzothiazoline-6-sulfonic acid) ABTS (Sigma^®^ A1888, St. Louis, MO, USA) with 2.45 mM of potassium persulfate, K_2_S_2_O_8_ (Sigma 216224) for 16 h in the dark at room temperature [23]. Following the method described by Choque et al., the obtained solution was diluted to an absorbance of 0.70 ± 0.02 (734 nm) using milliQ water or 95% ethanol. Therefore, two working solutions were obtained: ABTS–water and ABTS–ethanol [6].

Measurement of antioxidant activity of CL-NGP extract: The antioxidant activity of CL-NGP extract was expressed as mM (mmol per Litre) of vitamin C equivalent (VCEAC) using an ascorbic acid standard curve and measuring a 1/100 dilution of the extract in contact with 900 µL of ABTS–water. After 6 min of reaction between the sample and the ABTS–water solution, the absorbance was measured by UV spectrophotometry at 734 nm. The IC50 value was determined as the NGP concentration which produces an inhibition of 50% of ABTS^•+^ radicals and it was calculated by a calibration curve using CL-NGP extract. Measurements were performed in triplicate.

Determination of antioxidant capacity of bioactive films: for the determination of the antioxidant capacity of the films, 10 mg of each film were placed in Eppendorf tubes with 1 mL of ABTS–water or ABTS–ethanol. The absorbance of the solution in contact with the film was measured at different times: 30, 60, 90, 180, 300 and 900 s and the discoloration of the solution due to the activity of the antioxidant was recorded. Each condition was observed in duplicate. The results were expressed as a percentage of radical inhibition (% *RSA*) relative to each time through Equation (3):

(3) $$RSA\;\left(\%\right)=\frac{\left(Abs_{c}-Abs_{s}\right)}{Abs_{c}}\times 100$$

where $Abs_{c}$ is the absorbance of the control or blank (ABTS solution without film) and $Abs_{s}$ is the absorbance of the sample.

#### 2.4.7. Specific Migration Test in Fatty Food Simulant

The study of the release of the active compound from the films was carried out by determining the specific migration into 95% *v*/*v* ethanol as a fatty food simulant (FFS). Strips with dimensions of 6 cm^2^ of each film were completely immersed in 10 mL of simulant; in this way, the ratio of 6 dm^2^/L of simulant established in the European Standard 13130-2005 [24] was maintained. The migration test of the CL-NGPs was carried out at 25 and 40 °C and analysed at 120 and 240 h. All trials were performed in triplicate. After the incubation time, the strips were removed and the amount of CL-NGPs released to the simulant was quantified by UV spectroscopy at 280 nm [14]. A calibration curve was prepared using standard solutions of CL-NGPs in ethanol 95% *v*/*v*. Results were expressed as mg of CL-NGPs/L simulant. In addition, the antioxidant capacity of the CL-NGPs released to the simulant was evaluated using the ABTS method, described in Section 2.4.6.

#### 2.4.8. Water Sorption Isotherms

Water sorption isotherms were determined gravimetrically at 22 °C according to the standard procedure previously described [25]. Dried films of superficial area of 58 cm2 were placed in desiccators and equilibrated at different water activities aw (aw = %r.h./100). For this, saturated solutions of NaOH, MgCl_2_, NaBr, NaCl, and BaCl_2_ were used to generate conditions of aw of 0.10, 0.33, 0.57, 0.75, and 0.90, respectively. Dried atmospheres were obtained using silica gel. Samples were periodically weighed using an analytical balance (±10^−4^ g), and the evolution to equilibrium at each moisture condition was monitored until constant weight. The water content or hydration h, given in g of water per g of d.m. was evaluated as a function of aw. Experiments were performed in triplicates. Isotherms were fitted using the Guggenheim–Anderson–De Boer (GAB) model [26] through Equation (4):

(4) $$h\left(a_{w}\right)=\left(Ncka_{w}\right)/\left[\left(1+\left(c-1\right)ka_{w}\right)\left(1-ka_{w}\right)\right]$$

where *N* is the monolayer water content (g of water per g of dried mass) related to the number of primary binding sites of water molecules, *c* is a parameter related to the difference between the chemical potential of the water molecules in the upper layers and in the monolayer, and *k* a factor related to the difference between the chemical potential of the water in the pure liquid state and in the upper layers. *c* can also be interpreted as related to the force of the water-binding to monolayer and *k* as the capability of water binding to multilayer [27].

#### 2.4.9. Water Vapour Permeability Measurements

Experimental water vapour permeability of films was measured using the cup method described in ASTM-E96-2016 with some modifications [18,28]. Films were sealed on the top of cups containing a saturated salt solution of BaCl_2_ providing the highest r.h. of 90%. Test cups were placed in 7 L desiccators maintained at a constant temperature of 22 °C and 10% r.h. provided by a saturated salt solution of NaOH. Therefore, water vapour flux was determined from the weight loss of the cup. A fan was used to maintain uniform conditions inside the desiccators over the films [29]. Weight loss measurements were taken by weighing the test cup using an analytical balance (±10^−3^ g). Weight loss m (g) versus time t was plotted and when the steady state (straight line) was reached, 24 h further were registered. The experimental water vapour permeability coefficient *P_w_^exp^* was calculated as displayed in Equation (5):

(5) $$P_{w}{}^{exp}=\left(\frac{1}{A}\frac{\Delta m}{\Delta t}\right)\frac{L}{\Delta p_{w}}$$

where *A* = 2.2 × 10^−3^ m^2^ is the effective area of exposed film, Δ*m*/Δ*t* is the slope of a linear regression regarding weight loss versus time, *L* (m) is the film thickness, Δ*p_w_* = (*p_w_*_2_ − *p_w_*_1_) is the differential water vapour pressure across the film, and *p_w_*_2_ and *p_w_*_1_ are the partial pressures (Pa) of water vapour at the film surface inside and outside the cup, respectively. *P_w_^exp^* is given in units of g s^−1^ m^−1^ Pa^−1^. Experiments were performed in triplicate.

#### 2.4.10. Determination of Effective Water Solubility and Effective Water Diffusion

In biopolymeric films with a continuous and homogenous matrix, the water transport did not occur through pores but employing the mechanism of sorption-diffusion-desorption [30]. Therefore, the water vapour permeability depends on the hydration or water solubility in the film as well as the mobility of water in the matrix. Indeed, for hydrophilic biopolymeric films, the experimental water vapour permeability *P_w_^exp^* can be written as Equation (6) [31]:

(6) $$P_{w}{}^{exp}=D_{w}{}^{eff}\times S_{w}{}^{eff}$$

where *S_w_^eff^* (g m^−3^ Pa^−1^) is the effective water solubility coefficient over the concentration range *c_w_*_2_ to *c_w_*_1_, corresponding to water vapour pressure *p_w_*_2_ and *p_w_*_1_, respectively, and *D_w_^eff^* (m^2^ s^−1^) is the effective value of the diffusion coefficient over the water concentration range, *c_w_*_2_ to *c_w_*_1_.

Water sorption isotherms were used to estimate the water concentration *cw* (*cw* = *h*(*a_w_*) × *ρd.f.*) of each film specimen surface in the permeability experiment. *S_w_^eff^* value corresponding to pressure gradient or aw interval [*a_w_*_2_ = 0.9; *a_w_*_1_ = 0.1] was obtained by Equation (7):

(7) $$S_{w}{}^{eff}=\left[\left(h\left(a_{w2}\right)-h\left(a_{w1}\right)\right)/\left(p_{w2}-p_{w1}\right)\right]\times \rho_{d.f.}$$

where *h*(*a_w_*_2_) and *h*(*a_w_*_1_) are the water content of the film (g H_2_O per g d.m.) at its underside surface at *p_w_*_2_ and its surface outside the cup at *p_w_*_1_, respectively. According to Equation (6), *D_w_^eff^* was then calculated by Equation (8):

(8) $$D_{w}{}^{eff}=P_{w}{}^{exp}/S_{w}{}^{eff}$$

#### 2.4.11. Statistical Analyses

All results are shown as means with standard deviation. Statistical analyses were performed using OriginPro 8 (OriginLab Corporation, Northampton, MA, USA) and R software (v 3.4.4, R Foundation for Statistical Computing, Vienna, Austria). The data were subjected to the analysis of variance, and the means were compared by a post-hoc test (Tukey HSD). Differences were considered to be significant at *p* < 0.05. Errors of the GAB parameters obtained from the water sorption isotherms were estimated from the fit analysis.

## 3. Results and Discussion

### 3.1. Thermal Properties

**Figure 1:**
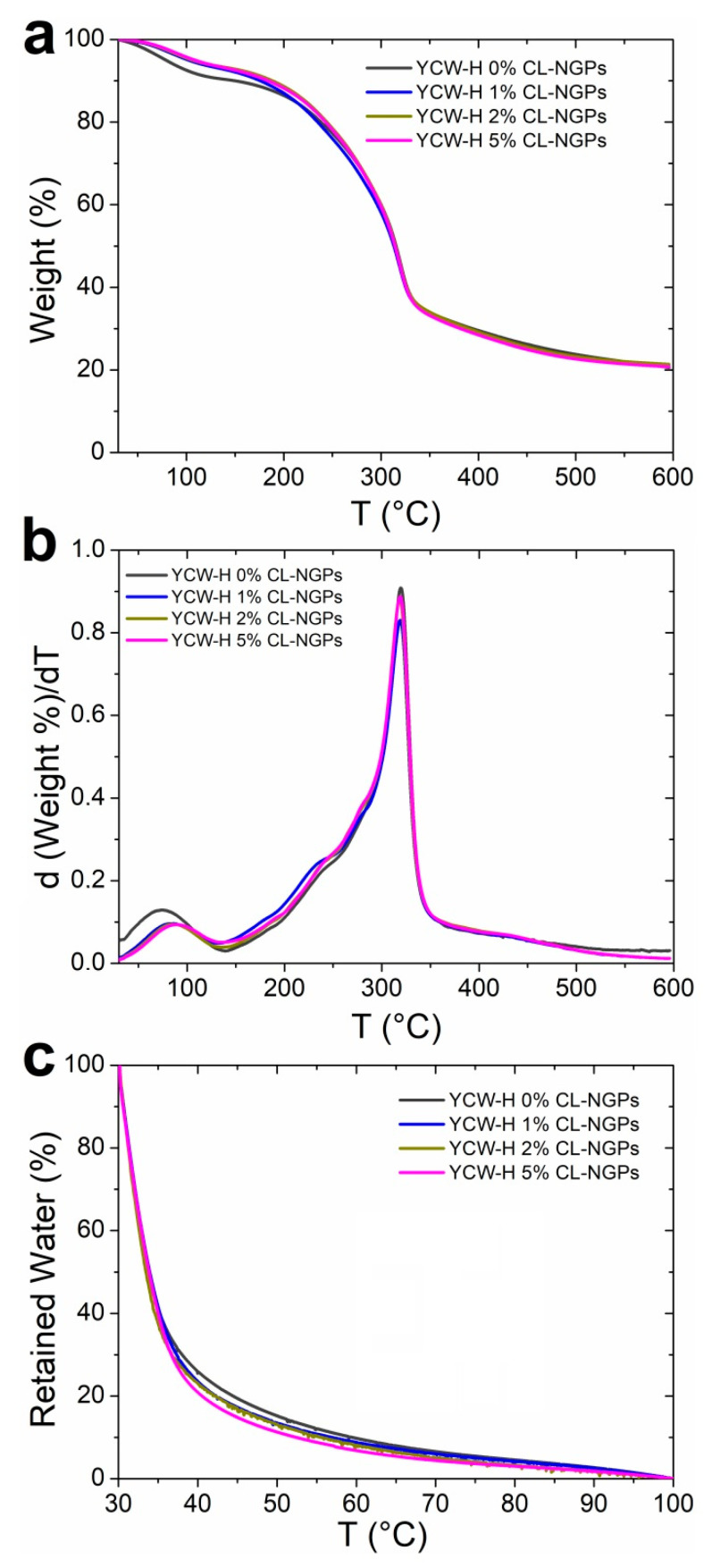
Thermogravimetric analysis of YCW-H films with different content of CL-NGPs: (**a**) percentage of mass loss; (**b**) derivative of the percentage of mass loss; and (**c**) percentage of retained water as a function of temperature.

Figure 1 shows the thermogravimetric analysis of the bioactive films that were previously conditioned at equilibrium in an atmosphere at 43% r.h.

The complete degradation between 30 and 600 °C is shown in Figure 1a. The first degradation zone up to 100 °C was attributed to water evaporation or dehydration. It is possible to say from Figure 1a that since all films were conditioned at the same r.h., films with the extract seemed to incorporate less water than the control during the conditioning process. This situation could be due to the hydrophobic characteristics of CL-NGPs, previously reported [6].

The thermal degradation of the YCW-H films presented a multi-stage profile. The mass loss up to 130 °C is due to the moisture elimination and possible volatilization of low molecular weight compounds [32]. A shoulder was observed at temperatures between 230 and 250 °C due to the degradation of the plasticizer and the peak at 306 °C, corresponding mainly to the degradation of polysaccharides (β-glucan) and massive degradation of cell wall proteins [18]. The initial degradation temperatures (T_ini_) did not show differences with the CL-NGPs addition, as well as in the maximum degradation temperature (T_max_) (see Figure 1b).

Figure 1c shows in detail the mass loss up to 100 °C due to dehydration. As seen in Figure 1c, the percentage of retained water values was lower with increasing amounts of CL-NGPs present in the film, indicating that the force of the hydration water molecules binding to the matrix decreases with increasing concentration of CL-NGPs.

### 3.2. Fourier Transform Infrared Spectroscopy (FTIR)

**Figure 2:**
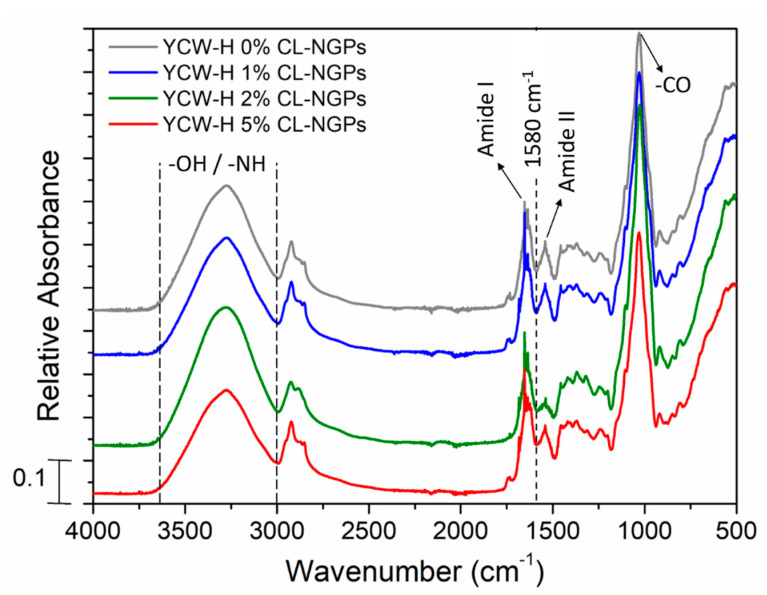
FTIR spectra of YCW-H films with different content of CL-NGPs. The spectra are normalized to the major peak at 1030 cm^−1^ for clarity.

The molecular interactions of the samples were studied by FTIR. The YCW-H film spectra showed no differences with the addition of CL-NGPs (Figure 2). In FTIR spectra of the system, bands in the range 3600–3000 cm^−1^ were associated with the absorption of hydroxyl groups (-OH) from wastewater, glycerol and polysaccharides and amino group (-NH) from proteins. The characteristic peaks of amide I and II were also observed at 1670 and 1550 cm^−1^, respectively. They demonstrate the presence of proteins (mannoproteins) in the matrix. Likewise, the main peak was obtained by carbon-oxygen (C-O) absorption of primary and secondary alcohols at 1100 cm^−1^, corresponding to glucose residues and β-linkages in the glucosidic chain [33].

No absorption bands characteristic of NGPs were identified, since they are mostly overlapped with the yeast cell wall spectrum. However, some differences were observed in the absorbance in those samples containing 2 and 5% CL-NGPs in the region of amide I and amide II, specifically at 1580 cm^−1^. This could be attributed to the presence of the NGP_S_ or the interaction between NGPs and the hydrophobic region of proteins from the yeast matrix [34,35].

### 3.3. Mechanical Uniaxial Tensile Tests

The results of the mechanical tests for the YCW-H samples are shown in Table 1. The mechanical properties of YCW-H films were affected by the addition of CL-NGPs depending on the concentration used. The addition of 1% of CL-NGPs produced a plasticization effect, where E value decrease and ε% increased. Then, TS and E increased with 2% of CL-NGPs, maybe due to the strong interaction of polymer chains because of the presence of the hydrophobic NGPs. This behaviour was also observed in sodium caseinate films plasticized with glycerol and added with different concentrations of tung oils [35]. These authors observed that by increasing the concentration of oil in the matrix from 5 to 15%, both E and TS increased due to heterogeneity in the system producing a protein-rich zone, increasing their aggregation and therefore their interaction. With the addition of 5% of CL-NGPs, it was observed that the TS and E decreased as compared to the control sample and the other concentrations of CL-NGPs. Excessive addition of a hydrophobic compound could induce the development of a heterogeneous matrix with the presence of discontinuous areas, producing points of failure. This same behaviour was obtained in polysaccharide films containing high concentrations of hydrophobic additives such as cocoa butter or soybean oil [36]. Additionally, elongation decreased in films with YCW-H 5% CL-NGPs, possibly because the addition of heterocyclic compounds from NGPs might prevent the free movement of polymer chains. A similar effect was observed in gelatin-based films with yucca extracts where elongation values were lower than the films without the extract because the hydrophobic tetracyclic structure of extract´s compounds did not allow conformational variations [37].

The addition of natural extract in biodegradable films based on biopolymers such as proteins and/or polysaccharides might present different effects on the mechanical properties of these matrices. Some authors have described that the addition of natural extracts could enhance or weaken tensile characteristics since different interactions between the additive and the matrix could exist, such as crosslinking, formation of heterogeneous biphasic structures or different structural arrangements of the components [38].

### 3.4. Colour Determination (CIELab Coordinates)

The CIELab coordinate values for YCW-H samples with CL-NGPs are shown in Table 2.

When analysing the L*, a decrease in these values was observed with the addition of increasing concentrations of CL-NGPs, demonstrating the development of darker films. The a* parameter also decreased with the addition of CL-NGPs but the b* parameter increased. Both results mark the tendency toward a yellow–green colour with the incorporation of CL-NGPs. Then, values of ∆*E* > 6 indicated that colour differences were visible in active materials when they were compared to control.

### 3.5. Measurement of Antioxidant Activity of the CL-NGP Extract by the ABTS Method

Table 3 shows the concentration of NGPs and the antioxidant capacity of the extract. The CL-NGP extract was measured in terms of VCEAC. It has already been shown that the NGP extract has antioxidant activity and, therefore, can be a good alternative to be used as a natural antioxidant agent in the preparation of bioactive films [6,14]. This work also demonstrated the antioxidant capacity of the obtained extract, as shown in Table 3. Although its antioxidant capacity in terms of IC50 is seven times lower than that of vitamin C (IC50: 2.50 mg/L), this is a satisfactory result when compared to the activity of other natural antioxidants such as essential oil from Ocimum basilicum (IC_50_: 687 mg/L) and Thymus algeriensis (IC_50_: 896 mg/L) [39] and other secondary metabolites produced by endophytic fungi (IC_50_: 54.45 mg/L) [40]. For reference, the values of IC_50_ measured by the ABTS method for α-tocopherol and BHA were 52.64 mg/L and 19.86 mg/L, respectively [41]. Therefore, the antioxidant capacity of CL-NGPs is significantly superior to these two compounds and showed promising characteristics for incorporation into the films.

### 3.6. Determination of Antioxidant Capacity of Bioactive Films

**Figure 3:**
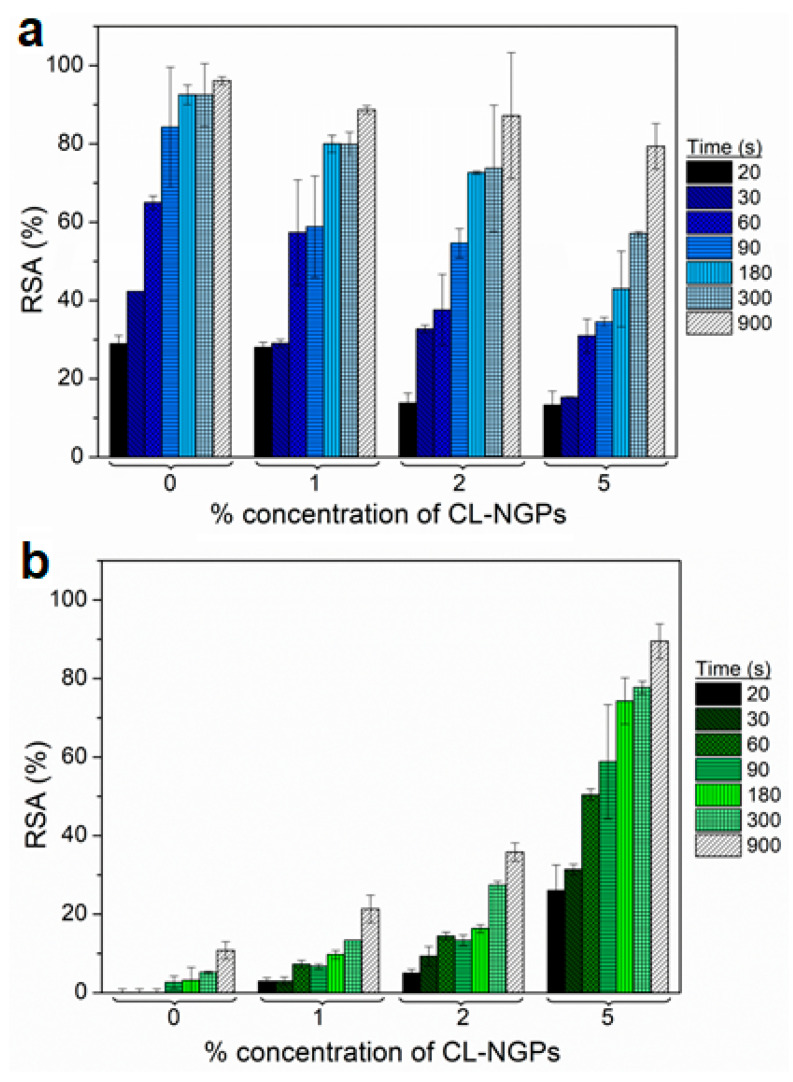
Antioxidant capacity of YCW-H films with CL-NGPs in: (**a**) ABTS–water solution and (**b**) ABTS–ethanol.

The results of the antioxidant capacity (% *RSA*) of the YCW-H films with the addition of CL-NGPs are shown in Figure 3a,b, over time in ABTS–water and ABTS–ethanol, respectively. It was observed that the % *RSA* values were higher in aqueous solution due to the reaction of tyrosine and β-glucans with ABTS–water [6]. Pérez-Jiménez and Saura-Calixto reported that samples with tyrosine and tryptophan, even at low concentrations, could produce overestimated results when the antioxidant capacity is analysed by the ABTS method in water [42]. The ABTS–water assays showed a lower antioxidant capacity of the samples with 2 and 5% CL-NGPs at short times due to a major CL-NGPs-matrix interaction than CL-NGPs-ABTS reaction. This effect at the final test time was cancelled, leaving the reactive groups exposed to the solvent.

When using the ABTS–ethanol solution, the interference of the film components was not observed, therefore it suggests that the antioxidant activity of the films was due to the CL-NGPs. It was observed that for all formulations with CL-NGPs, the antioxidant capacity increased over time, demonstrating a gradual release of the additive in the ABTS–ethanol solution. Furthermore, the formulation that showed greater effectiveness was developed with 5% CL-NGPs obtaining values close to 90% *RSA* after 15 min of testing.

### 3.7. Specific Migration Test in Fatty Food Simulant

**Figure 4:**
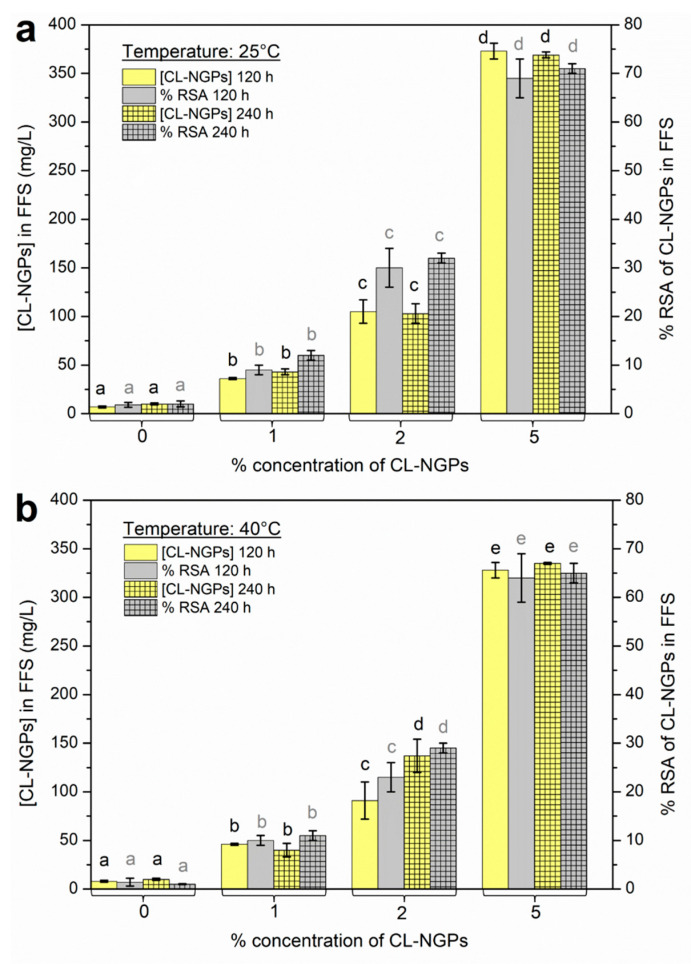
Concentration values (mg/L) and antioxidant capacity (% *RSA*) of migrated CL-NGPs in the fatty food simulant (FFS) (ethanol 95% *v*/*v*) at: (**a**) 25 °C and (**b**) 40 °C. The different letters assigned in each column (black colour for quantification of CL-NGPs and grey colour for and antioxidant capacity of CL-NGPs) refer to statistical significant differences (*p* ≤ 0.05).

Following the proposed methodology, once the incubation time was over, the simulant was analysed. The quantification of the released CL-NGPs was performed using the calibration curve using different concentrations of CL-NGP extract. Figure 4 shows the concentration values and the antioxidant capacity of migrated CL-NGPs in the fatty food simulant (FFS) at 25 °C (Figure 4a) and 40 °C (Figure 4b). For both temperatures, the concentration of migrated active agent increased with the initial content of CL-NGPs in the films for the same time, indicating a dependency on the initial concentration of the analyte [3]. An increase in antioxidant capacity was observed for formulations with higher CL-NGP content. Radical inhibition superior to 50% was observed at both temperatures with the addition of 5% CL-NGPs. These results correspond appropriately with a major concentration of CL-NGPs in the simulant.

### 3.8. Hydration and Water Vapour Transport Properties

**Figure 5:**
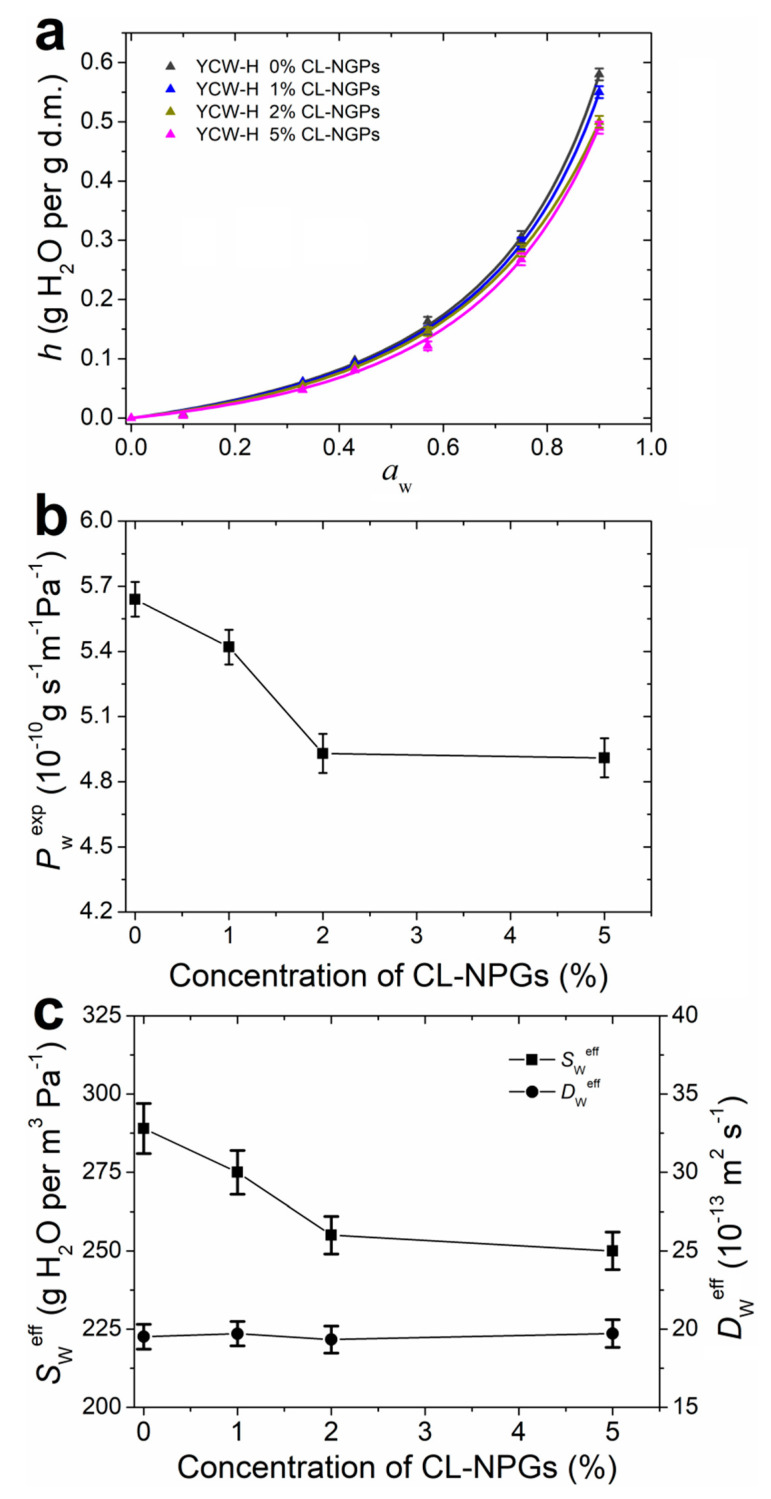
Hydration and water vapour transport of YCW-H films with different content of CL-NGPs. (**a**) Water sorption isotherms. Experimental data were fitted with Equation (4) and fitted parameters shown in Table 4. (**b**) Experimental water vapour permeability *P_w_^exp^* as a function of the concentration of CL-NGPs. (**c**) Effective water solubility Sweff and effective water diffusion coefficient *D_w_^eff^* as a function of the concentration of CL-NGPs.

Biopolymers such as polysaccharides and proteins interact strongly with water, and therefore the films formed from these macromolecules are hydrophilic materials [5]. Figure 5a shows the water sorption isotherms of the YCW-H films with the addition of CL-NGPs. Experimental points were fitted with the GAB model (Equation (4)) and fitted parameters are shown in Table 4.

A significant increase in the hydration of the films can be observed in Figure 5a for aw >0.6, suggesting that most of the hydration water molecules were forming multilayers and were indirectly bound to the polymeric matrix. This behaviour is common in most biopolymeric films [5]. Therefore, hydration water molecules in biopolymeric materials are likely to be moved by diffusion mechanism through a water gradient concentration [31]. Otherwise, as the CL-NGP content of the samples increased, the water content at the same aw was lower.

Table 4 displays the parameters obtained from fitting the sorption isotherms with the GAB model. Parameter N, related to the number of primary binding sites, and parameter c, linked to the force of the water-binding to primary sites, showed a slight tendency to decrease with the addition of CL-NGPs. Moreover, parameter k decreased significantly, indicating that the amount of bound water in the hydration multilayer decreased with the increase in the concentration of CL-NGPs.

Water sorption isotherms of samples with 2 and 5% of CL-NGPs showed a significant decrease in hydration due to the hydrophobicity of the additive (Figure 5a). Possibly, the addition of CL-NGPs acts as a barrier to the incorporation of hydration water due to its hydrophobicity. The addition of active compounds might increase the interactions between the polymer chains thus decreasing free volume and, therefore, reducing the number of hydration water molecules. However, at high concentrations (5% CL-NGPs), the antioxidant compounds could interact with each other through hydrophobic interactions without increasing the interactions between the polymer chains, and consequently, no significant variation in hydration was observed as compared to YCW-H 2% CL-NGPs.

Hydration water affects the main structural and functional properties of hydrophilic films because water acts as a plasticiser by embedding itself between the polymer chains [43]. Moreover, hydration water is related to the water vapour transport through hydrophilic polymeric films because it depends on the hydration or water solubility in the film as well as the mobility of water in the matrix [18,31]. Studies on the water vapour transport through the films are necessary to optimize the barrier properties required during its applications.

Experimental water vapour permeability *P_w_^exp^* of YCW-H films as a function of the concentration of CL-NGPs is shown in Figure 5b. It is possible to observe a marked decrease in *P_w_^exp^* with the addition of 2% CL-NGPs, which remained constant with the addition of 5%. Equation (6) allowed the evaluation of water solubility and diffusion as separate phenomena that contribute to *P_w_^exp^*. Figure 5c shows the effective water solubility *S_w_^eff^* and the effective water diffusion *D_w_^eff^* with respect to CL-NGP concentration. It can be observed that as the concentration of NGPs increased, *S_w_^eff^* behaved in the same way as *P_w_^exp^*, while *D_w_^exp^* remained constant. As a consequence, the water vapour barrier properties of YCW-H films supplemented with NGPs were given by the behaviour of *S_w_^eff^* because *D_w_^exp^* was not altered by the NGP content. These results were consistent with those obtained in the water sorption isotherms. As already explained, at concentrations of up to 2% CL-NGPs, the hydrophobicity of the compound improved the water vapour barrier properties of the films. At higher concentrations, antioxidant compounds could interact with each other, without modifying the interactions between the polymer chains, and therefore *S_w_^eff^* was not modified as compared to YCW-H 2% CL-NGPs.

## 4. Conclusions

In the present work, it was demonstrated that it is feasible to obtain active films based on yeast cell wall using homogenization as a cell breakdown process and with the addition of CL-NGPs as antioxidant compound. The thermogravimetric and spectroscopic study revealed that the samples were composed of wastewater, plasticizer, β-glucans and mannoproteins. Colour was significantly changed with the addition of the extract and the mechanical properties of the samples were consistent with possible polymer–polymer interactions within the presence of active compound. The extract of CL-NGPs was shown to have antioxidant activity and this is maintained after incorporation into biopolymer matrices. Food simulation migration studies confirmed that CL-NGPs migrated in fatty food simulants and was effective in terms of their antioxidant capacity. Furthermore, the addition of the extract enhanced the material’s water vapour barrier properties due to its hydrophobic nature. The results obtained in this work showed that bioactive yeast cell wall-based films containing CL-NGPs have the potential for use as packaging material in systems of interest for the food industry. In addition, the application of these materials could be extended to other fields, such as pharmaceuticals and medicines.

## Figures and Tables

**Table 1:** Mechanical parameters of YCW-H films with CL-NGPs.

Samples	E (MPa)	ε (%)	TS (MPa)
YCW-H 0% CL-NGPs	37 ± 3 ^a^	32 ± 3 ^a^	4.5 ± 0.7 ^a^
YCW-H 1% CL-NGPs	24 ± 3 ^b^	42 ± 2 ^b^	4.4 ± 0.3 ^a^
YCW-H 2% CL-NGPs	40 ± 2 ^a^	45 ± 3 ^b^	5.6 ± 0.1 ^b^
YCW-H 5% CL-NGPs	24 ± 2 ^b^	29 ± 1 ^a^	3.5 ± 0.3 ^c^

The different letters assigned in each column refer to significant differences (*p* ≤ 0.05).

**Table 2:** CIELab coordinate values for YCW-H films with CL-NGPs aggregate.

Samples	L*	a*	b*	Δ*E*
YCW-H 0% CL-NGPs	90 ± 1 ^a^	−0.2 ± 0.1 ^a^	10 ± 1 ^a^	-
YCW-H 1% CL-NGPs	86 ± 1 ^b^	−2.8 ± 0.1 ^b^	26 ± 1 ^b^	16 ± 1 ^a^
YCW-H 2% CL-NGPs	83 ± 1 ^c^	−4.3 ± 0.1 ^c^	36 ± 2 ^c^	27 ± 2 ^b^
YCW-H 5% CL-NGPs	79 ± 2 ^d^	−4.6 ± 0.9 ^c^	52 ± 2 ^d^	45 ± 7 ^c^

The different letters assigned in each column refer to significant differences (*p* ≤ 0.05).

**Table 3:** Concentration of NGPs and antioxidant capacity values of the extract. (Vit C: vitamin C; N.C.: not concerned).

Extract	% of NGPs	VCEAC (mM)	VCEAC (mg vit C/g NGPs)	IC50 (mg/L)
CL-NGPs	90	13.47	130.98	17.78
Vit C	N.C.	N.C.	N.C.	2.50

**Table 4:** Values of the GAB parameters fitted for the water sorption isotherms of films displayed in Figure 5a. h_90%r.h_. refers to the hydration equilibrium value at 90% r.h. The reported values of the statistical parameter R^2^ indicate a very good acceptance of the fit model. Errors are estimated from the fit analysis.

Samples	N (g/g)	c	k	h_90%r.h._ (g/g)	R^2^
YCW-H 0% CL-NGPs	0.23 ± 0.01	0.8 ± 0.1	0.84 ± 0.02	0.58 ± 0.01	0.999
YCW-H 1% CL-NGPs	0.22 ± 0.01	0.7 ± 0.1	0.82 ± 0.01	0.55 ± 0.01	0.999
YCW-H 2% CL-NGPs	0.20 ± 0.01	0.6 ± 0.1	0.78 ± 0.2	0.50 ± 0.01	0.999
YCW-H 5% CL-NGPs	0.20 ± 0.01	0.6 ± 0.1	0.77 ± 0.1	0.49 ± 0.01	0.999
